# Comparative assessment of amino acids composition in two types of marine fish silage

**DOI:** 10.1038/s41598-021-93884-4

**Published:** 2021-07-27

**Authors:** Mukund Gauthankar, Rakhee Khandeparker, Mamatha S. Shivaramu, Komal Salkar, Rayadurga Anantha Sreepada, Mandar Paingankar

**Affiliations:** 1 Biological Oceanography Division, CSIR–National Institute of Oceanography (CSIR–NIO), Dona Paula, Goa 403004 India; 2 Food Protectants and Infestation Control Department, CSIR–Central Food Technological Research Institute (CSIR–CFTRI), Mysuru, Karnataka 570020 India; 3 Department of Zoology, Government Science College, Gadchiroli, Chamorshi Road, Gadchiroli, Maharashtra 442605 India

**Keywords:** Biochemistry, Ecology, Zoology, Environmental sciences

## Abstract

Fish silage is a brown liquefied product achieved by the action of enzymes when finely grounded whole/parts of either single or mixed fish types are subjected to acidification. This study made a comparative assessment of biochemical and nutritive properties, especially the amino acid composition in supernatant phase of formic acid silages prepared from two fish types, Indian mackerel (*Rastrelliger kanagurta*) and false travely (*Lactarius lactarius)* representing fat fish (FF, fat content > 5%) and lean fish (LF, fat content < 5%), respectively during 35 days of fermentation (DoF). Significantly higher content of total amino acid (TAA) and free amino acids (FAA) were recorded in FFS (TAA, 41.2 ± 0.03 mg/g; FAA, 31.3 ± 0.003 mg/g) compared to LFS (TAA, 35.8 ± 0.07 mg/g; FAA, 18.26 ± 0.003 mg/g; FAA, 31.3 ± 0.003 mg/g) (*p* < 0.05). At the end of 35 DoF, the concentrations of amino acids such as asparagine, histidine, isoleucine, valine, cysteine, serine, lysine and arginine were significantly higher in FFS as compared to LFS. The relative amino acid composition of FFS and LFS varied in accordance with DoF and the relationship was found to be highly significant (ANOVA, *p* < 0.00001). High concentrations of l-amino acids such as leucine, glutamic acid and arginine were recorded in both FFS and LFS. In conclusion, the analysis suggested that a fermentation period of 25–30 days showed a significant effect on the composition of amino acids in both types of ensilage compared to other fermentation periods (*p* < 0.05). Considering the role of amino acids in enhancing the plant growth and proliferation, the findings of the present study are quite useful.

## Introduction

Globally, fish represents about 17% of animal protein supply and 7% of all protein for human consumption^1^. The processing of fish entails to significant removal of parts of the fish, such as heads, fins, bones, guts, etc. which can represent between 30 and 70% of the fish^2^. Waste of such high magnitude requires proper disposal to prevent detrimental environmental consequences such as eutrophication in water bodies^3^. On the other hand, fishery biowaste has potential to generate high value-added compounds, such as hydroxyapatite, collagen, enzymes, proteins and fish oils etc^4^. Therefore, many valuable components can be sourced from fishery biowaste including proteins, collagen, proteases and variety of other metabolites instead of dumping into the environment^5^.

Production of fish silage from discards is an excellent alternative for extracting biomolecules such as proteins, amino acids, lipids, fatty acids etc^4,5^. The use of fishery waste as a major source of nitrogen for the cultivation of seaweeds has been documented^6^. Crop yields obtained by using fish silage as fertiliser at par with those reported using traditional fertiliser has also been reported^7–9^. Owing to its high content of hydrolysed proteins (free amino acids and peptides), fish silage has been reported to enhance the growth performance in fish^2^. The significance of amino acids in promoting plant growth, food and dietary supplement has been widely documented^10–14^. The role of amino acids aiding many cellular processes in plants such as proliferation, intracellular pH regulation, energy generation and enhancing the plants defence against various environmental stresses have been highlighted^15,16^. Amino acids, particularly leucine, serine and proline are involved in cell signalling process, while others act as precursors for phytohormonal biosynthesis^17–19^. Furthermore, amino acids are known to exhibit a chelating effect on micronutrients and enhanced absorption and transportation of micronutrients inside the plant have been reported when applied in conjunction with fertilisers^16^.

An important role played by free amino acids (FAA) and short peptides in plant nutrition have been documented^20^. By virtue of their smaller size compared to complex protein structures, the direct absorption of FAAs by plants via roots or stromal openings stimulating better plant growth has also been highlighted^20,21^. The apparent advantages such as simple, inexpensive and safety of preparation and most importantly its nutritionally rich composition render the fish silage a better alternative as a plant growth promoter^22^. Considering the most of the previous studies focussed on the heterogeneous fishery waste, the precise role of fish type in influencing the nutritional profile, more specifically, total amino acids (TAA) and free amino acids (FAA) has not been elucidated.

With this background, a study to assess the changes in biochemical constituents, particularly the composition of TAA and FAA in supernatant phase in two types of fish ensilages, FFS (> 5% fat by weight) and LFS (< 5% fat by weight) during 35 DoF was conducted. It is anticipated that the results of the study would help in the formulation of low-cost foliar spray for enhancing the plant growth by efficiently using the fish biowaste.

## Results and discussions

### Degree of hydrolysis

Organic silages prepared from fat fish (FFS) and lean fish (LFS) had a characteristic tawny brown colour which was accompanied with a strong characteristic salty-fishy odour. At the end of 5 DoF, both FFS and LFS exhibited sluggish liquefaction which increased progressively concomitant with the DoF (Table S1). Liquefaction is an indicator of tissue hydrolysis due to the action of acid. During 35 DoF, the degree of hydrolysis (measured in terms of liquefaction volume) increased progressively with the DoF in both types of ensilages and was relatively higher in LFS compared to FFS on all sampled DoF (Table S1). In general, lipolysis supersedes the proteolysis in all major biochemical processes^23^. A relatively higher degree of hydrolysis recorded in LFS may be attributed to the presence of a greater proportion of light muscles compared to dark muscles. Relatively greater susceptibility of light muscles to hydrolysis compared to dark muscles might be due to lower lipid content in the former^23^.

Irrespective of fish type, the measured pH values in both types of ensilages (FFS and LFS) were similar (data not shown) and the values showed a progressive increase from 1.0 ± 0.03 (0 DoF) to 6.0 ± 0.03 (35 DoF). Such an increasing trend in pH with the advancement in DoF could be attributable to gradual solubilisation of boney material with the advancement fermentation time^24–26^.

### Changes in principal biochemical constituents

During the 35 DoF, the concentrations of total protein (TP) in both FFS and LFS progressively increased with the DoF and showed significant differences with the advancement of DoF (*p* < 0.05) (Table 1). Irrespective of ensilage type, the measured concentrations of TP (FFS, 97.82 ± 0.01 mg/mL; LFS, 62.96 ± 0.06 mg/mL) on 30 DoF were significantly higher (*p* < 0.05) than those concentrations recorded on other DoF (Table 1). In contrast, Ramasubburayan et al. (2013)^27^ reported a slight decrement in TP content (~ 4%) on 30 DoF fish silage. Similarly, TP concentrations in FFS on 30 DoF were significantly higher than in LFS on corresponding DoF (*p* < 0.05).

**Table 1:** Changes in the concentrations (mg/mL) of total protein (TP), total carbohydrate (TC) and total lipid (TL) in supernatant of two silages prepared from fat fish (FFS) and lean fish (LFS) during 35 days of fermentation (DoF). Values (mg/mL) are mean ± SE.

DoF	TP (mg/mL)		TC (mg/mL)		TL (mg/mL)	
	FFS	LFS	FFS	LFS	FFS	LFS
10	42.19 ± 0.03^a^	38.75 ± 0.07^a^	6.71 ± 0.02^ab^	2.56 ± 0.003^abc^	110.10 ± 0.04^ab^	102.54 ± 0.02^ab^
15	53.40 ± 0.03^ab^	48.02 ± 0.06^ab^	6.84 ± 0.02^ab^	3.52 ± 0.03^ab^	134.96 ± 0.01^ab^	131.55 ± 0.02^ab^
25	61.71 ± 0.03^ab^	54.44 ± 0.07^ab^	5.55 ± 0.03^ab^	3.85 ± 0.04^ab^	164.85 ± 0.02^ab^	81.77 ± 0.03^abc^
30	97.82 ± 0.01^bc^	62.96 ± 0.06^bc^	5.83 ± 0.03^ab^	3.43 ± 0.04^ab^	200.98 ± 0.03^bc^	109.85 ± 0.04^ab^
35	52.95 ± 0.03^ab^	49.01 ± 0.07^ab^	5.71 ± 0.02^ab^	3.56 ± 0.03^ab^	159.25 ± 0.03^ab^	114.68 ± 0.02^ab^

Different superscript letters of biochemical constituent in the same column and paired comparison between FFS and LFS indicate significant difference between DoF (*p* < 0.05).

Although the total carbohydrate (TC) content was observed to be significantly higher in FFS compared to LFS on all DoF (Table 1), the differences, however were insignificant (*p* > 0.05). The TC content was significantly higher (*p* < 0.05) in FFS on 30 DoF than estimated from LFS on the corresponding DoF. Furthermore, the significant differences in TC concentrations with DoF in both types of fish silages were not discernible. In addition to contributing as an energy source for the growth and metabolism of plants, the carbohydrates are known to shield plants against abiotic stressors such as osmotic imbalance and salinity^28^. In the absence of previous reports, the concentrations of TC recorded in the present study would serve as baseline information.

The total lipid (TL) content in FFS was relatively higher compared to LFS on all DoF (Table 1). It has been reported that the TL content of fish silage vary in accordance with the fat content of the fish species^29^. Compared to other DoF, the concentrations of TL in FFS and LFS were significantly higher on 25 DoF and 30 DoF, respectively (*p* < 0.05). TL content in LFS did not follow any particular trend during 35 DoF (Table 1). On the other hand, concentrations of the TL in FFS progressively increased from 110.10 ± 0.04 mg/mL (10 DoF) to 200.98 ± 0.03 mg/mL (30 DoF) and dropped to 159.25 ± 0.03 mg/mL (35 DoF). This is in agreement with previous studies by Palkar et al.^30^. Such an increase in the TL concentrations with the progress of the DoF until 35 DoF, particularly in FFS may be due to considerable release of stored lipid during the liquefaction of the fish tissue^31^. Furthermore, a substantial decrease in TL content in FFS on 35 DoF could be attributable to oxidation of lipid due to presence of higher proportion of long chain polyunsaturated fatty acids compared to LFS^32^. By virtue of crucial role played by lipid components in plants in providing structural integrity to the cell membrane, cell signalling related to biotic as well as protection from abiotic stressors and in mediating a variety of cellular metabolic reactions^33^, a detailed study on fatty acids profiles in fish silage thus becomes of paramount importance.

### Comparative assessment of total amino acid composition

**Figure 1:**
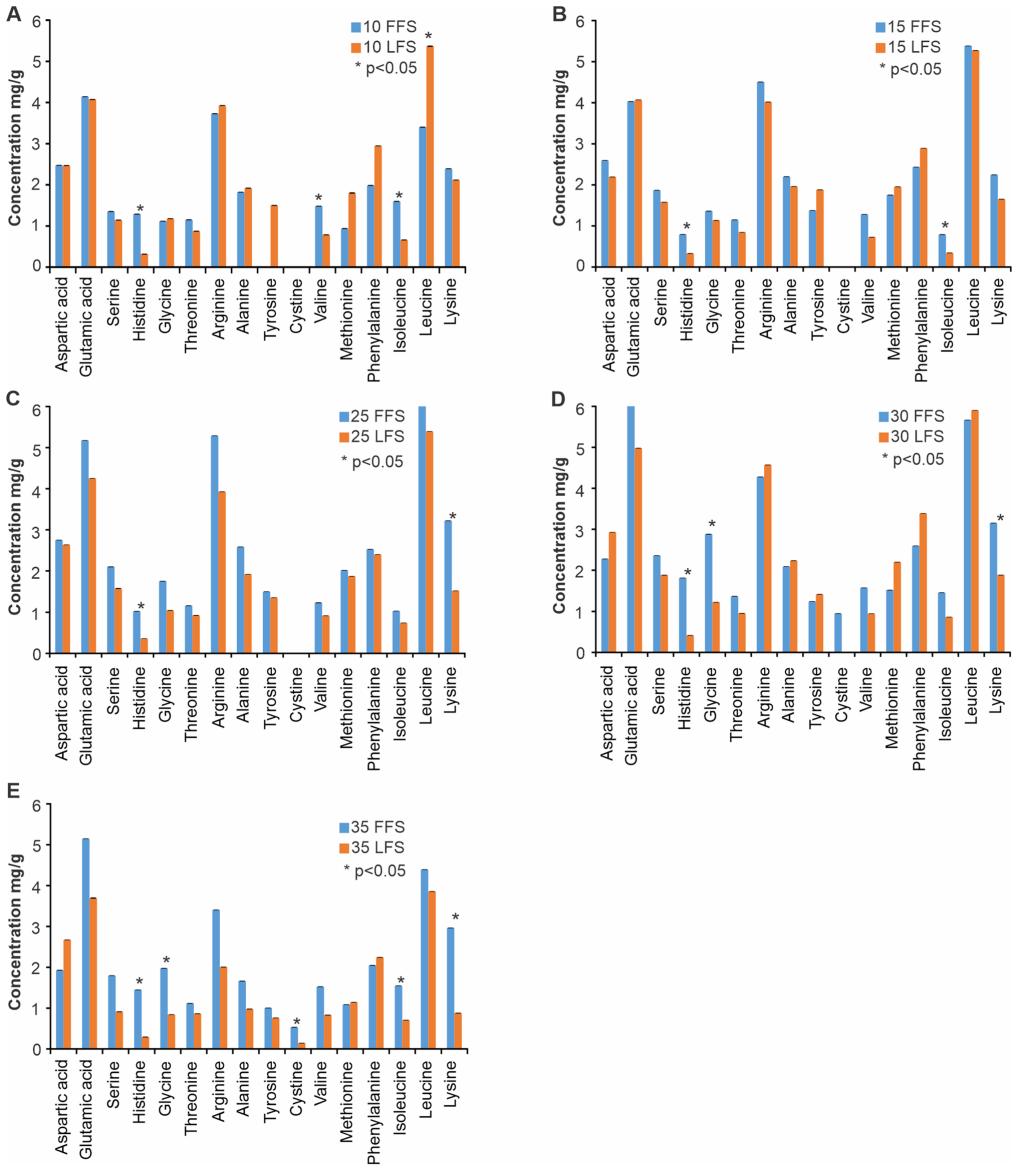
Composition of total amino acids (mg/g) in two types of fish ensilages (FFS—fat fish silage; LFS—lean fish silage) during 35 days of fermentation (DoF). Data are mean ± SD. * p < 0.05.

Although the content of total amino acids (TAA) was relatively higher in FFS than in LFS during 35 DoF however, their profiles were almost similar (Fig. 1). The TAA content in FFS peaked on 30 DoF (41.2 ± 0.033 mg/g) from a lowest value of 28.9 ± 0.033 mg/g (10 DoF) and then dropped to 33.6 ± 0.033 mg/g (35 DoF) (Fig. 1). The profiles of TAA in FFS on 30 DoF in terms of concentration (mg/g) followed the order: glutamic acid and leucine (6.0 ± 0.033) > Arginine (5.3 ± 0.033) > lysine (3.2 ± 0.033) > glycine (2.9 ± 0.033) > phenylalanine (2.6 ± 0.033) > serine (2.4 ± 0.033) > aspartic acid (2.3 ± 0.033) > alanine (2.1 ± 0.033) > histidine (1.8 ± 0.033) > valine (1.6 ± 0.033) > methionine (1.5 ± 0.033) > isoleucine (1.5 ± 0.033) > threonine (1.4 ± 0.033) > cysteine (0.946 ± 0.033).

On the other hand, content of TAA in LFS also followed a similar trend with the concentrations peaking on 30 DoF (35.8 ± 0.066 mg/g) from an initial concentration of 31.1 ± 0.066 mg/g (10 DoF) (Fig. 1). The concentrations of profiles (mg/g) of TAA in LFS on 30 DoF followed the order, leucine (5.90 ± 0.033) > glutamic acid (4.97 ± 0.033) > arginine (4.5 ± 0.033) > phenylalanine (3.38 ± 0.033) > aspartic acid (2.92 ± 0.033) > alanine (2.23 ± 0.033) > methionine (2.19 ± 0.033) > lysine (1.882 ± 0.033) > serine (1.881 ± 0.033) > tyrosine (1.410 ± 0.033) > glycine (1.219 ± 0.033) > threonine (0.953 ± 0.033) > valine (0.945 ± 0.033) > isoleucine (0.864 ± 0.033) > histidine (0.417 ± 0.033).

A comparative assessment of profiles of TAA in both FSS and LFS during all DoF revealed a similar pattern, albeit with obvious differences in the concentration of few amino acids (Fig. 1). It has been hypothesised that the occurrence of decarboxylation that follows transamination of amino acids as a consequence of increase in pH during fermentation is known to cause a decrement in the concentration of few amino acids, especially valine and isoleucine^34^. During the present study, the concentrations of histidine, valine, isoleucine, glycine and lysine were significantly higher (*p* < 0.05) in FFS compared to LFS (Fig. 1). The plausible explanation for lower levels of isoleucine and valine in LFS may be due to transamination of these amino acids^34–36^. The reduction in the concentration of few amino acids during the fermentation attributable to the differential chemical reactions between alpha and aldehyde groups of amino acids has been highlighted^31,34–36^. In contrast, a striking similarity in the composition of TAAs in fish silages prepared from a variety of raw materials with or without addition of molasses has been reported^34–36^. Therefore, it does appear that concentrations of few TAA during the fermentation are directly dependent on the type of raw material and fermentation period.

In the present study, amino acids such as cysteine, tryptophan asparagine and glutamine, were conspicuously absent in both types of ensilages on all DoF. The usage of mild organic acid (formic acid) for the preparation of fish silage resulting in the partial and or complete destruction of cysteine has been reported^37.^  Absence of tryptophan in both FFS and LFS during the 35 DoF could directly be linked to its highly unstable nature in acidic medium, thus rendering it to become the first limiting amino acid in formic acid-based fish silages^38^. The increase in pH concomitant with the DoF enhancing the rate of transamination and protease-based degradation of TAAs such as asparagine and glutamine might explain the absence of these amino acids^39^.

### Content and composition of free amino acids

**Figure 2:**
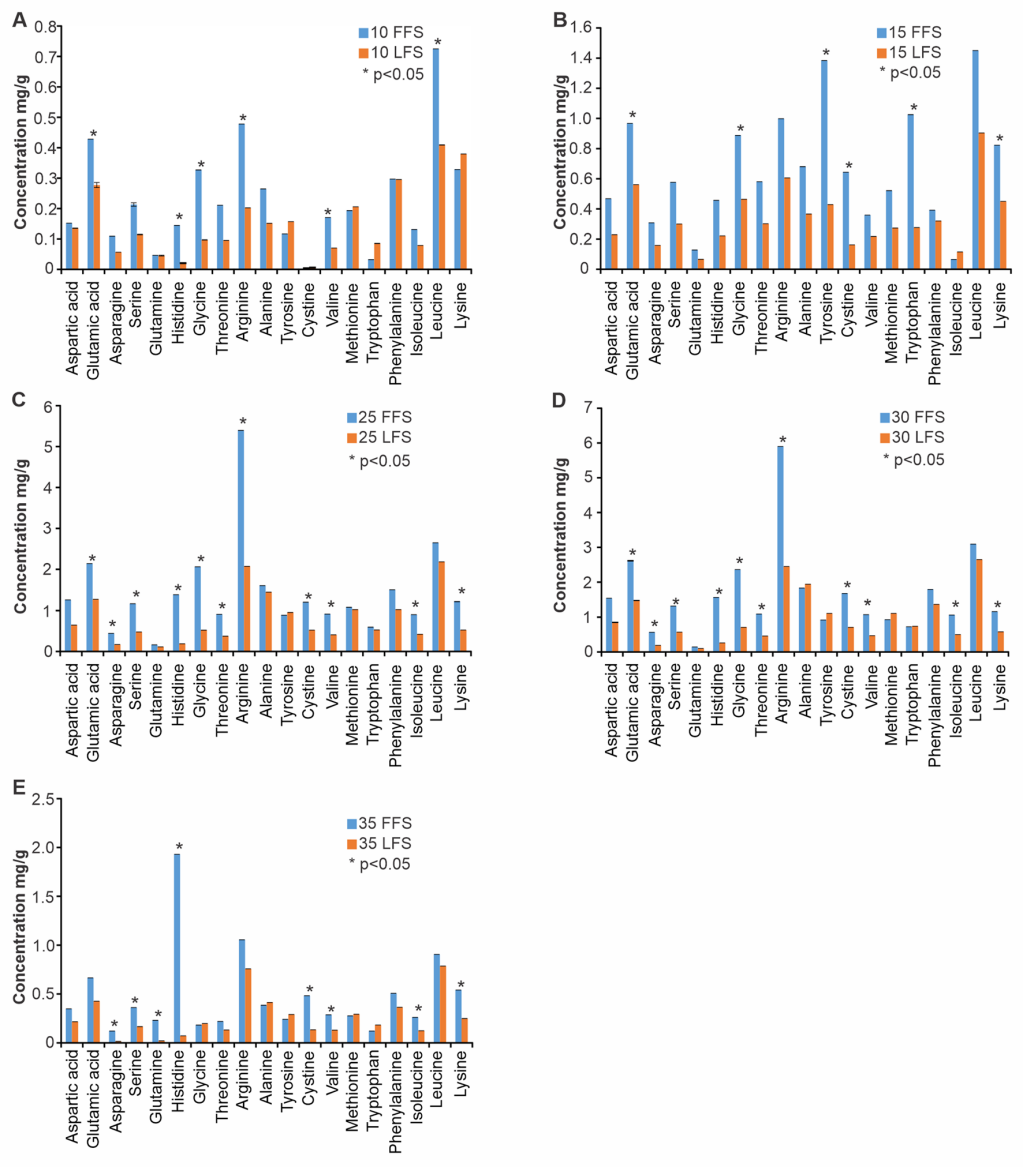
Composition of free amino acids (mg/g) in two types of fish ensilages (FFS—fat fish silage; LFS—lean fish silage) during 35 days of fermentation (DoF). Data are mean ± SD. * p < 0.05.

During the 35 DoF, the content of FAA in both types of fish silages followed the trend of TAA (Fig. 2). In FFS, the levels of FAA increased from 4.37 ± 0.003 mg/g (10 DoF) and attained a highest concentration of 31.35 ± 0.003 mg/g on 30 DoF (Fig. 2). The concentrations (mg/g) of 18 FAAs in FFS on 30 DoF followed the order: arginine (5.90 ± 0.003) > leucine (3.09 ± 0.003) > glutamic acid (2.61 ± 0.003) > alanine (1.83 ± 0.003) > phenylalanine (1.79 ± 0.003) > cysteine (1.67 ± 0.003) > histidine (1.56 ± 0.003) > aspartic acid (1.54 ± 0.003) > serine (1.32 ± 0.003) > lysine (1.16 ± 0.003) > threonine (1.09 ± 0.003) > valine (1.07 ± 0.003) > isoleucine (1.06 ± 0.003) followed by methionine (0.93 ± 0.003) > tyrosine (0.92 ± 0.003) > tryptophan (0.72 ± 0.003) > asparagine (0.57 ± 0.003) > glutamine (0.15 ± 0.003).

Profiles of FAA in LFS also followed a similar trend that of FFS. Concentrations (mg/g) of a sum of 19 FAAs peaked on 30 DoF (18.26 ± 0.003) which was almost 6 times higher than the content recorded on 10 DoF (2.87 ± 0.003). In contrast, the highest recorded amino acid in LFS was leucine (2.65 ± 0.003) followed by arginine (2.45 ± 0.003), alanine (1.94 ± 0.003), glutamic acid (1.47 ± 0.003), phenylalanine (1.37 ± 0.003 m), tyrosine (1.11 ± 0.003), methionine (1.11 ± 0.003), aspartic acid (0.85 ± 0.003), tryptophan (0.74 ± 0.003), glycine (0.71 ± 0.003), cysteine (0.71 ± 0.003), lysine (0.58 ± 0.003), serine (0.57 ± 0.003), isoleucine (0.50 ± 0.003), valine (0.47 ± 0.003), threonine (0.46 ± 0.003), histidine (0.26 ± 0.003), asparagine (0.19 ± 0.003) and glutamine (0.10 ± 0.003).

### Changes in amino acid composition during fermentation

The relative composition of FAAs in FFS and LFS varied in accordance with DoF, and the relationship was found to be highly significant (ANOVA, F value = 12.72; *p* < 0.00001). The amino acid profiles in LFS and FFS during 35 DoF were dominated by arginine, glycine, methionine, glutamic acid, phenylalanine, lysine and leucine (Fig. 2). At the end of 10 DoF, the concentrations of dominant amino acids such as arginine, glutamic acid, glycine, valine and leucine were significantly higher in FFS than in LFS (Mann–Whitney test, *p* < 0.05). Concentrations of tyrosine, lysine, glycine, glutamic acid were markedly higher in FFS than in LFS at the end of 15 DoF (Mann–Whitney test, *p* < 0.05). Furthermore, the concentrations of asparagine, histidine, threonine, valine, isoleucine, serine, glycine, cysteine, lysine, glutamic acid, arginine were significantly higher in FFS as compared to LFS at the end of 25 DoF and 30 DoF, respectively (Mann–Whitney test; *p* < 0.05) (Fig. 2). At the end of 35 DoF, the concentrations of amino acids such as asparagine, histidine, isoleucine, valine, cysteine, serine, lysine and arginine were significantly higher in FFS compared to LFS.

**Figure 3:**
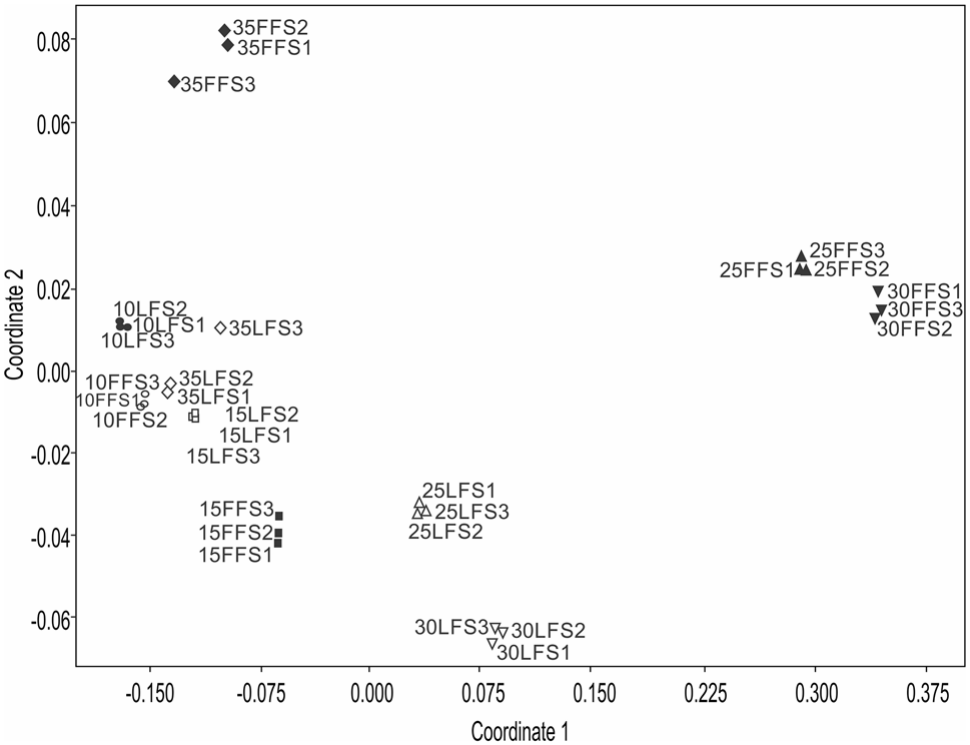
Non-metric multidimensional scaling (nMDS) (Bray–Curtis similarity) of log-transformed free amino acid content in two types of ensilages ((FFS and LFS) at different days of fermentation (DoF).

Results of the one-way analysis of similarities (ANOSIM) test and the non-metric multidimensional scaling (nMDS) plot (Fig. 3) indicated that the differences in the amino acid composition in two types of ensilages (FFS and LFS) during the 35 DoF were highly significant (Fig. 3; nMDS, stress 0.032, one-way ANOSIM R = 0.9881; *p* < 0.0001). The dissimilarity in amino acid composition between two types of ensilages (FFS and LFS) at the end of 10 DoF was mainly contributed by leucine, arginine, glycine, glutamic acid and histidine (Table 2). At the end of 15 DoF, tyrosine, tryptophan, leucine, cysteine, glycine and glutamic acid were majorly responsible for the dissimilarity observed between the two types of ensilages (FFS and LFS). A significant dissimilarity in amino acid composition between two types of ensilages (FFS and LFS) at the end of 25 DoF and 30 DoF is mainly attributable to arginine, glycine, histidine, glutamic acid. At end of 35 DoF, histidine, arginine, cysteine, lysine, glutamic acid were the main amino acids that contributed to the dissimilarity in amino acid composition between two types of fish ensilage (FFS and LFS) (Table 2). This spatial segregation suggested that fermentation period was majorly responsible for the dissimilarity in altering the amino acid profiles between two types of fish ensilages (FFS and LFS). Overall, the analyses suggest that fermentation period of 25‒30 days exerted a profound effect on the composition of amino acids in both types of ensilage compared to other fermentation periods (*p* < 0.05) (Table 2).

**Table 2:** Similarity percentage analysis (SIMPER) showing average dissimilarity percentages of most common amino acid in two types of ensilages at the end of different days of fermentation (DoF).

Days of fermentation (DoF)	Free amino acids		
	Arginine	Glycine	Glutamic acid
10 DoF	3.81	3.15	2.23
15 DoF	2.05	2.17	2.11
25 DoF	7.92	3.65	2.06
30 DoF	6.83	3.32	2.45
35 DoF	2.86	0.10	1.46

The observed decline in TAA and FAA contents beyond 30 DoF could possibly be due to solubilisation of the bony materials which results in the production of amines as a consequence of an action by formic acid. Amines being alkaline, might have resulted in a drastic increase in pH of the silage with the progress of the DoF, eventually leading to lower yields of amino acids^40,41^. A drastic decrease in the concentration of amino acids with the progress of fermentation due to the formation of major biogenic amines (cadaverine, tyramine and ornithine) has been reported^42^. Furthermore, other amines such as histamine (from histidine), 2-phenylethylamine and tyramine (from phenylalnine and tyrosine decarboxylation, respectively), tryptamine from tryptophan, putrescine obtained from decarboxylation of arginine and cadaverine from lysine have also been reported to be responsible in decreasing the concentration of amino acids in fish silages^43–47^.

The transformation of FAAs into biogenic amines via decarboxylation, oxidative deamination or by other undefined pathways during the extended fermentation has been reported to be the major factor responsible for the reduction in the content and altering the composition of amino acids^48^. Örlygsson (1995)^49^ attributed the reduction in the levels of alanine as a consequence of the Stickland reaction, wherein alanine gets converted to acetic acid upon reaction with glycine and water. By-products of this reaction (ammonia and carbon dioxide) may render further increase in the pH and thus advancing the degradation of FAA to biogenic amines and eventually resulting in decreased FAA levels.

### Amino acids as plant growth promoters

Amongst all FAAs, the levels arginine, leucine and glutamic acid were highest during 25‒30 DoF, particularly in FFS (Fig. 2). In addition to its role as nitrogen storage and transport in many plants, arginine also promotes the synthesis of phytohormones that are responsible for fruiting and flowering of the plants^50^. Amino acids such as glycine and glutamic acid help in the development of plant tissue and synthesis of chlorophyll. Acting as an osmotic agent in the cytoplasm, L-glutamic acid aids in the opening of stomata. Similarly, leucine is actively involved in plant defence against environmental stressor and also in protecting the reproductive tissues of plants from pathogens^51^. The enzymatic locus in plants involved in protein synthesis can only recognise the L-form of amino acids^52^. L-form of amino acids (methionine, tryptophan and proline) acting as precursors of phytohormones (ethylene, espermine and espermidine)—involved in inhibition of senescence^53–55^, energy generation and plants' defence against various environmental stresses^18,19^. Another phytohormone, auxin involved in promoting cell elongation, phototropism, root and shoot elongation^56^ and pollen fertility protection of plant from water stresses is produced from tryptophan^57^ The enzymatic locus involved in protein synthesis in plants is capable of recognising the L-form of amino acids^52^. The fish silages produced during this study were rich in L-amino acids, especially the free amino acids could be an inexpensive and excellent alternative as a plant growth enhancer or as an organic fertiliser for boosting crop yields.

Untreated fishery waste has potential to trigger eutrophication consequent to its release into the coastal marine waters. Transforming this biowaste into a useful product could minimise both environmental and disposal problems. An application of organic foliar formulations derived from fish silage on okra (*Abelmoschus esculentus*) and red amaranth (*Amaranthus tricolor*) resulting in enhanced plant growth, leaf pigmentation and greater yields in comparison to that of the chemical fertilizer has been demonstrated^8^. Furthermore, effectiveness of liquid fish silage (concentration, of 5–10%) as an organic fertiliser for the growth of pakchoy (*Brassica rapa* L. subsp*. chinensis*) has been documented^9^. Results of the present study points out that fish biowaste can be transformed into an eco-friendly supplementary plant growth enhancer due to the high yield of free amino acids. A comparative assessment of amino acids (both total and free) in silages produced from two different fish types based on the fat content with different days of fermentation forms the novelty of the present study.

## Conclusions

In conclusion, the results highlighted that a duration of 25–30 days of fermentation (DoF) yielded maximum content of TAA and FAA in both FFS and LFS as evidenced by similarity percentage (SIMPER) analysis. Although the composition of FAA in FFS and LFS was almost similar, individual concentrations of FAA, however differed significantly. The fish silages produced from two types of fish proved to be rich in L-amino acids, especially the free amino acids thus making it better, cheaper, most efficient, easy to process and administer as an alternative plant growth enhancer or as an organic fertiliser. The nutritional quality of the formulated silage could be gauged from its tawny brown colour with characteristic fishy odour corresponding to optimal quality (up to 30 DoF), while the blackish colour silage with stale fishy odour representing degradation stage (> 35 DoF).

Owing to their low molecular weight and short-length peptides, free amino acids from fishery waste could easily be assimilated by roots and stromata of plants. Future follow-up e*x-situ* studies focussing on fish silage in enhancing the growth and metabolism of plants is expected to delineate the precise role of micronutrients present in fish silages. Furthermore, comparative studies focusing on mixed fishery waste varying proportions of FF and LF may provide greater insights into the different types of amino acids for target-oriented application.

## Methods

### Collection of fish and preparation of silage

The Indian mackerel (*Rastrelliger kanagurta)* and false travely (*Lactarius lactarius*), representing as fat fish (FF, > 5% fat by weight) and lean fish (LF, < 5% fat by weight), respectively were selected for the preparation of silages, FFS and LFS. The fishes discarded (fish unfit for human consumption, but not yet decomposing) were collected from the fish market, Panaji, Goa (India) (Lat. 15.5002 ºN; Long. 73.8223 ºE) and transported to the laboratory in ice for the preparation of silage.

The waste generated from cleaning process of two types of fishes (FF and LF) were ground separately in a heavy-duty grinder (SUMEET Appliances Pvt. Ltd. India) until paste-like consistency was achieved. The finely minced fish paste (fish type-wise, 50 g each) was then carefully dispensed into separate sterile narrow-mouth conical flasks (250 mL). This was followed by addition of 3% (1.5 mL) of formic acid (EMSURE, 98–100% Merck Life Science, Finland-USA). The experimental flasks silage type-wise (in triplicate) were cotton-plugged and kept at room temperature (26 ± 1 ºC) following the method described by Arruda et al. (2007)^58^. Altogether 30 ensilage flasks representing 15 flasks each for FFS and LFS in triplicate for each designated days (10, 15, 25, 30 and 35) of fermentation (DoF).

### Sample collection and biochemical analysis

During the 35 DoF, the flasks were swirled twice a day (0900 h and 2100 h) for 5 min to maximise the action of the acid. The immediate mixture was of semi-solid in consistency (0 DoF) which, eventually turned to semi-liquid (15 DoF) and finally transformed into an almost liquid state on 30 DoF as result of acid hydrolysis. On each designated DoF (0, 10, 15, 20, 25, 30 and 35 DoF), the resultant liquefied silages (FFS and LFA) were carefully decanted into fresh polypropylene centrifuge tubes for determining the volume of liquefaction, measurement of pH and estimation of major biochemical constituents and amino acid composition (TAA and FAA).

Considering the role of pH in influencing the degree of hydrolysis, the pH measurements in both types of silage (FFS and LFS) were made on 10, 15, 25, 30 and 35 DoF using calibrated pH meter (EUTECH pH meter, Model No. pH 700). Prior to pH measurements, the fish silage flasks were mixed thoroughly by vigorous handshaking. The glass electrode of pH meter was inserted into the flasks containing silage in such a way that the bulb of the electrode was fully immersed inside the silage without touching the bottom of the flask. Final values were noted when the readings stabilised, and for consistent readings, the measurements were repeated (at least thrice). Before each measurement, the electrode was thoroughly rinsed with deionised water to remove any particles adhering to the electrode. In the case of FFS flasks, before taking the pH measurements, the top thin layer of oil was removed carefully with the help of pipette in order to avoid interference.

### Estimation of principal biochemical constituents

For estimation of principal biochemical constituents, total crude protein (TP), total carbohydrate (TC) and total lipid (TL) in FFS and LFS, the supernatant fraction was used. In order to separate out the solid and liquid phases, the liquefied ensilages (FFS and LFS) were transferred carefully into clean polypropylene tubes and centrifuged at 7500 rpm for 20 min (EPPENDORF, 5430R). Concentrations of TP, TC and TL in silage samples (in triplicate) were estimated by following standard procedures. The total protein content (TP) was analyzed using Lowry’s Folin-Ciocalteu method with bovine serum albumin as the standard^59^. Total carbohydrate (TC) was estimated using the phenol–sulphuric acid method^60^. Total lipid (TL) content was estimated by extracting samples of fish silage in a mixture of chloroform–methanol following Folch's method^61^. The extracts were dried by evaporation under a stream of nitrogen and the weight (TL) was determined.

### Composition and amino acid content

Briefly, the estimation of amino acid (TAA and FAA) content in FFS and LFS involved two stages, the sample preparation followed by the analysis of chromatographic data^62^. The content of TAAs and FAAs in two types of ensilages (FFS and LFS) were carried out following the method described by Henderson & Brooks (2010)^63^ using Agilent 1260 Infinity System (Agilent Technologies Inc., USA).

The HPLC system is equipped with a quaternary pump, an auto-sampler, column compartment and diode array detector. The analysis were performed using Agilent ZORBAX Eclipse plus C18 Column (4.6 × 250 mm with 5 µm packing particle, Agilent Technologies Inc., USA). For derivatization of the primary amino acids,.O-phthalaldehyde was used The mobile phase, A comprises 10 mM Na_2_HPO_4_: 10 mM Na_2_B_4_O_7_: 5 mM NaN_3_ (pH 8.2), whereas, the mobile phase, B comprises of the mixture containing acetonitrile: methanol: water in the ratio of 45:45:10 (v:v:v). During the separation process, a flow rate of 1.5 mL/min was maintained. The primary amino acids in the samples were quantified by using alpha-aminobutyric acid as an internal standard^62,63^.

For the quantification of d TAA content, a an aliquot (100 µL) of sample was hydrolysed with 2 mL of 6 N HCl (EMPARTA ~ 36%, Merck Life Science Pvt. Ltd., India) in amber-coloured vial. The sample vials were spiked with 200 µL of internal standard (10 nm/µL ABA, Sigma-Aldrich, USA), purged with N_2_ gas (2 m) , screw-capped and then kept at 110 °C for 24 h^34^.

For extraction of FAA in FFS and LFS, the samples were first treated with 15 mL of 10% (w/v) ice-cold trichloroacetic acid (≥ 99.0% purity, Sigma-Aldrich, Germany) to facilitate the partial unfolding of the protein. The mixture was then extracted in equal volumes of di-ethyl ether (99.9% purity, HiMedia, India**)**. To achieve the maximum recovery of FAAs, this procedure was repeated five times. The aqueous layer thus collected was evaporated to dryness (< 40 °C) in a rotary vacuum evaporator (ROTEVA EQUITRON).

Post hydrolysis, the supernatants of FFS and LFS were neutralised by addition of 2 mL of 6 N NaOH and dried in a rotary vacuum evaporator. Dried samples were reconstituted in 2 mL of deionised water and injected into HPLC system (Agilent 1260 Infinity, USA for understanding the composition of TAA. For analysis of FAA profiles, the dried samples were reconstituted in 25 mL of deionised water and 1 µL was injected into the HPLC system. Each quantified amino acid was expressed as mg/g^63^.

### Statistical analysis

To examine the significant variability in the composition of amino acids in two types of ensilages (LFS and FFS) during the 35 DoF, both similarity-based multivariate analyses (SIMPER and nMDS) and univariate analysis (ANOVA) were employed. Composition of amino acid was subjected multivariate analysis with a nMDS based on Euclidean similarity matrix using the software PAST version 4.02^64^. The nMDS was carried out in conjunction with one-way ANOSIM, to test whether clusters in the nMDS plot differed significantly from each other. Finally, SIMPERanalysis was performed with logarithmically transformed percentages to determine the main amino acids contributing to dissimilarity in two types of ensilages and DoF. The assumptions of normality and homoscedasticity were checked with Shapiro–Wilk and Levene test prior to ANOVA. Kruskal–Wallis test for equal medians, Mann–Whitney sequential Bonferroni correction and Tukey’s pairwise Dunn's post-hoc correction were performed for post-hoc pairwise comparisons using 95% confidence limits.

## Supplementary Information

Supplementary Information.

## Data Availability

All data are fully available without restriction. All relevant data are within the manuscript and its Supporting Information files.
