# Supplementary material for: Comparative assessment of amino acids composition in two types of marine fish silage
Source: Sci Rep. 2021 Jul 27;11:15235. doi: 10.1038/s41598-021-93884-4 (PMC8316558; doi:10.1038/s41598-021-93884-4)
Supplement: Supplementary file 1 — Supplementary Information. [file 41598_2021_93884_MOESM1_ESM.pdf]

Comparative Assessment of Amino Acids Composition in two types of Marine Fish Silage

Mukund Gauthankar[1], Rakhee Khandeparker[1], Mamatha S Shivaramu[1,2], Komal Salkar[1], Rayadurga Anantha Sreepada[1✉], Mandar Paingankar[3]

[1]Biological Oceanography Division, CSIR–National Institute of Oceanography (CSIR–NIO), Dona Paula, Goa–403004, India. [2]Food Protectants & Infestation Control Department, CSIR–Central Food Technological Research Institute (CSIR–CFTRI), Mysuru, Karnataka–570020, India. [3]Department of Zoology, Government Science College Gadchiroli, Chamorshi Road, Gadchiroli, Maharashtra–442605, India.

✉email: sreepada@nio.org

Table S1. Mean liquefaction volumes (mean ± SE) at the end of different days of fermentation (DoF) in two types of ensilages (FFS = fat fish silage; LFS = lean fish silage)

DoF	Mean liquefaction volume (mL)	
	FFS	LFS
10	7.833±0.166	10.00±0.00
15	8.50±0.288	13.660±8819
25	15.66±0.333	21.33±0.333
30	21.2±0.2000	24.33±0.333
35	22.33±0.333	26.33±0.166
